# Transfection of oral squamous cell carcinoma with human papillomavirus-16 induces proliferative and morphological changes *in vitro*

**DOI:** 10.1186/1475-2867-6-14

**Published:** 2006-05-22

**Authors:** Karl Kingsley, Devin Johnson, Susan O'Malley

**Affiliations:** 1 Department of Biomedical Sciences, University of Nevada, Las Vegas – School of Dental Medicine, 1001 Shadow Lane B315, Las Vegas, Nevada, 89106, USA

## Abstract

**Background:**

Human papillomavirus has been implicated in virtually all cervical cancers and is believed to be the primary etiological factor that transforms cervical epithelia. The presence of HPV in oral cancers suggests that HPV may play a similar role in transforming the oral epithelia. The prevalence of HPV in oral cancers is highly variable, however, presenting problematic issues regarding the etiology of oral cancers, which must be investigated more thoroughly. Past analyses of HPV in cancers of the oral cavity have largely been confined to retrospective studies of cancer patients. The purpose of this study was to examine the potential for HPV16 infection to alter the proliferative phenotype of oral squamous cell carcinoma *in vitro*.

**Results:**

This study found that the oral squamous cell carcinoma cell line, CAL27, transfected with HPV16, exhibited significantly increased proliferation, compared with non-transfected CAL27. The increased proliferation was observed under low density conditions, even in the absence of serum. Moreover, these effects were specific to proliferation, adhesion, and morphology, while cell viability was not affected.

**Conclusion:**

This study represents one of the first investigations of the effects of HPV16 infection on the proliferation, adhesion, and morphology of an oral squamous cell carcinoma cell line *in vitro*. The finding that HPV16 has the ability to measurably alter adhesion and proliferative potential is significant, indicating that HPV may have multiple influences on precancerous and cancerous lesions and should be explored as a risk factor and mediator of cancer phenotypes. These measurements and observations will be of benefit to researchers interested in elucidating the mechanisms of oral cancer transformation and the factors governing carcinogenesis and progression.

## Background

Human papillomavirus (HPV) has been implicated in many intraepithelial neoplasias and invasive squamous cell carcinomas [1-3]. The worldwide prevalence of HPV in cervical carcinomas has been determined to be as high as 99.7%, with HPV types 16 and 18 implicated most frequently [4,5]. Recent studies indicate that of these two high risk types of HPV, HPV16 is the most prevalent type found in oral cancers, most notably in oral squamous cell carcinomas (OSCC). These studies have also provided evidence that oral infection with HPV is a significant independent risk factor for OSCC, determining that HPV is detected in 46.5% of OSCC, compared with its detection in 10% of normal oral mucosa [6-10].

Why HPV is found in virtually all cervical cancers, but only in a subset of OSCC, has yet to be explained. The mechanism of HPV carcinogenesis has been well established for cervical cancers. Early studies have demonstrated that the HPV-expressed E6 and E7 proteins function concomitantly to disrupt the *p53 *and *Rb *tumor suppressor genes, regulators of cell-cycle checkpoints at the G_1 _phase [5,11,12]. These pathways have also been implicated in head and neck cancers [13], suggesting the plausible assumption that HPV may also play a similar and significant role in the development of oral cancers.

Much of the literature related to HPV infection and oral cancers involves epidemiologic studies and retrospective analyses of tumor biopsies and HPV infection in OSCC. Despite the correlative evidence of HPV infection with oral cancer, other investigations have discovered that HPV16 is rarely found in premalignant oral lesions, and may therefore not be necessary for progression of oral mucosa to malignancy [8,14,15]. The results of some studies even suggest that patients with certain HPV positive oral cancers seem to have greater survival rates than those with oral cancers that are negative for HPV [7,10].

While the aforementioned studies have informed our understanding of HPV prevalence in oral cancers and have begun to explain the mechanisms of HPV transformation, they have not adequately addressed the apparent contradictory evidence that HPV infection may not be causally related to the formation of all, or even most, oral carcinomas. For instance, although some studies have demonstrated the transformation of human foreskin and cervical keratinocytes *in vitro *using HPV16 [3,16], no studies thus far have sufficiently investigated the role of HPV in already transformed OSCC; in particular, no *in vitro *studies have established or defined the role of acquired HPV with alterations of the proliferative phenotype of OSCC.

One possible explanation for the presence of HPV DNA in oral cancer biopsies, but not in premalignant oral lesions, may be the inability of PCR and other detection techniques to distinguish between HPV infections causally related to cancer development and those that are concomitant, non-causal HPV infections [9,17]. By examining the effects of HPV16 infection on transformed OSCC *in vitro*, the etiologic factors that are necessary and sufficient to induce transformation of the oral mucosa, and those factors that may only promote proliferative potential in already transformed OSCC, can be elucidated.

The goal of this study was to examine the role of HPV16 infection in altering proliferative phenotype of OSCC *in vitro*. We hypothesized that HPV16 infection of the OSCC cell line, CAL27, would mediate phenotypic alterations of these cells, particularly adhesion, proliferation, and morphology. We tested this hypothesis by transfecting CAL27 with the full-length HPV16 genome and establishing cellular adhesion and proliferation assays for HPV16-transfected and non-transfected CAL27.

Our results provide one of the first demonstrations that HPV16 significantly affects the proliferative potential of CAL27 *in vitro*. Furthermore, HPV16 transfection produces measurable differences in adhesion and morphology in CAL27 cells, compared with non-transfected controls.

## Results

### Transfection and RT-PCR

**Figure 1:**
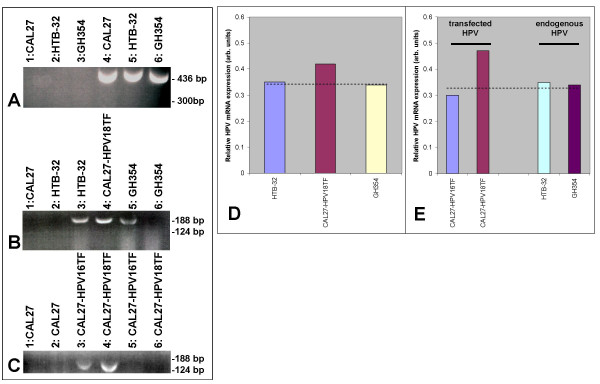
**CAL27 cells expressed HPV 16 *in vitro *only after transfection**. RT-PCR from total RNA demonstrated that CAL27, HTB-32, and GH354 cells did not express HPV16 mRNA (A: lanes 1–3) but did express laminin-5 γ2-subunit, the positive control (A: lanes 4–6). RT-PCR confirmed transfection efficiency using HPV18-vector (B: lane 4), compared to HPV18-/HPV+ cell lines, HTB-32 and GH354 (B: lanes 3, 5); no cell lines expressed HPV16-specific mRNA (B: lanes 1, 2, 6). CAL27-HPV16-transfectants expressed HPV16-specific mRNA (C: lane 3) but not HPV18-mRNA (C: lane 5) and CAL27-HPV18 transfectants expressed HPV18-specific mRNA (C: lanes 4) but not HPV16 (C: lane 6). CAL27 controls expressed neither HPV16 nor HPV18 mRNA (C: lanes 1, 2). Densitometry measurements of relative endpoint RT-PCR band intensities for endogenous HPV (GH354) were compared to HPV18 controls (D). CAL27 HPV-transfectant mRNA levels were compared with positive controls (E).

To confirm that CAL27 does not already harbor HPV16, we performed RT-PCR on total RNA isolated from cultured CAL27 cells using oligonucleotide primers specific for HPV16. In addition, two HPV16-negative cervical carcinoma cell lines, HTB-32 and GH354, were included as controls. Our results demonstrated that CAL27, HTB-32, and GH354 did not express HPV16 mRNA (Fig.1A: lanes 1–3). Primers specific for the γ2-subunit of the extracellular matrix (ECM) protein, laminin-5, were used as a positive control (Fig.1A: lanes 4–6).

We tested the effectiveness of the transient transfections of CAL27 with HPV18 by performing RT-PCR on CAL27-HPV18 and a cell line known to harbor endogenous HPV 18, GH354. The HPV18-negative cell line, HTB-32, was included as a control. Our results demonstrated that CAL27-HPV18 and GH354 expressed HPV18 mRNA (Fig. 1B: lanes 4–5). Our results also indicated, however, that HTB-32 cell line, purported to be HPV negative, expressed HPV18 mRNA (Fig. 1B: lane 3). Our results further verified the absence of HPV16-specific mRNA in these cell lines (Fig. 1B: lanes 1, 2, 6).

We completed transient transfections of CAL27 with HPV16 and with HPV18 subtypes. Our results indicated that CAL27-HPV16 transfectants expressed HPV16-specific mRNA and CAL27-HPV18 transfectants expressed HPV18-specific mRNA (Fig. 1C: lanes 3–4). Furthermore, we demonstrated that CAL27 does not express HPV16 or HPV18 mRNA (Fig. 1C: lanes 1–2) and that each transfection was specific for the HPV subtype (Fig. 1C: Lanes 5–6).

Densitometric measurements of RT-PCR band intensity for comparison of endogenous HPV to transient transfections revealed a 1.25-fold higher level of HPV expression in CAL27-HPV18 transfectants (Fig. 1D). Comparison of HPV16 and HPV18 CAL27 transfectants from subsequent experiments confirmed HPV18-specific mRNA expression was approximately 1.25-fold that of endogenous HPV mRNA expression, while HPV16-specific mRNA expression was roughly equivalent to endogenous HPV levels (Fig. 1E).

### Proliferation

**Figure 2:**
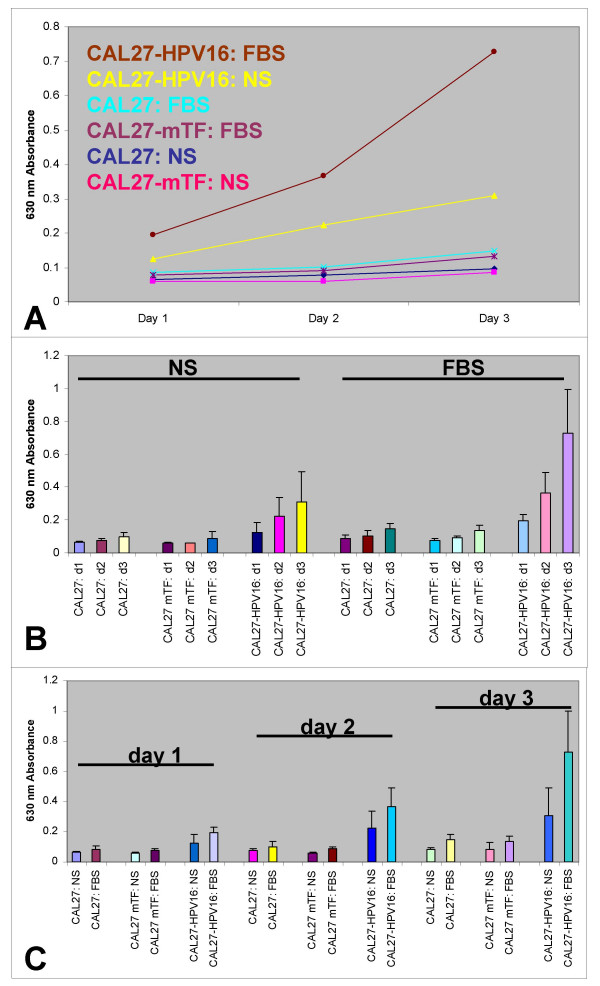
**HPV 16 increased CAL27 proliferation *in vitro***. CAL27 cells were plated at low density (10^4 ^cells per well) and allowed to proliferate with 10% fetal bovine serum (FBS) or no serum (NS). Proliferation of CAL27 HPV16-transfected cells increased significantly from day 1 to day 3 under FBS or NS treatment (A) (n = 96, p < .01). Direct comparison of NS with FBS treatment, day 1 – day 3, demonstrated effect of HPV16 on CAL27 proliferation (B) (n = 96, p < .01). Direct comparison of day 1, day 2 and day 3 results under NS and FBS revealed HPV16 increased CAL27 proliferation 3.2-fold under NS treatment and 5.0-fold under FBS (C). Three separate, independent replications of this experiment were performed (n = 24 wells per trial).

To test our hypothesis that HPV infection alters the proliferative potential of OSCC, we performed *in vitro *proliferation assays to determine the relative effects of HPV16 transfection on CAL27 cells. CAL27, CAL27 mock transfectants (mTF) and CAL27 transfectants (HPV16) were plated in media containing fetal bovine serum (FBS), as well as in media containing no serum (NS), and their proliferation was measured over three days in three separate, independent experiments. Our results demonstrated that CAL27-HPV16 cells proliferated at a significantly higher rate from day 1 – day 3, increasing by more than 5-fold over CAL27 when in FBS-containing media (n = 48, p < 0.01). Furthermore, CAL27-HPV16 cells were able to proliferate, even in the absence of serum, at a rate significantly higher than non-transfected CAL27 cells, increasing by more than 3.5-fold from day 1 to day 3 (n = 48, p < 0.01) (Fig. 2A).

CAL27 and CAL27-mTF cells exhibited similar proliferation in the presence and absence of serum, varying only in the degree to which the cells responded to the presence of serum. In the presence of serum (FBS), CAL27 and CAL-mTF cells proliferated by approximately 75% (1.75-and 1.72-fold, respectively) over three days and were statistically indistinguishable from each other (n = 48, p = .39). In the absence of serum (NS), CAL27 and CAL27-mTF cells increased in proliferation by approximately 40% (1.42-and 1.41-fold, respectively) and were also statistically indistinguishable (n = 48, p = .50). CAL27 and CAL27-mTF cells demonstrated an approximate increase of 35% in the FBS treatment compared with the NS treatment (1.75-fold increase in FBS vs. 1.4-fold increase in NS). CAL27-HPV16 cells increased their response in the presence of serum to a larger extent than did CAL27 non-transfected cells. Furthermore, proliferation of CAL27-HPV16 cells increased by 380% (3.8-fold) over three days under FBS treatment and by 260% (2.6-fold) in NS conditions. This represents a differential increase in FBS versus NS treatment of HPV16-transfected cells of more than 120% (Fig. 2B).

When the data were analyzed by trial day (day 1, day 2, day 3), and the differences in cellular proliferation between NS and FBS were compared within each experimental group (non-transfectants, mock transfectants, HPV-transfectants), another pattern emerged. The mean difference in cellular proliferation between NS and FBS for CAL27 and CAL27-mTF was 1.3-fold; a 30% increase under FBS conditions. This pattern remained constant on day 2 and increased slightly on day 3 (1.75-fold increase in FBS-versus NS-treated CAL27 cells, 1.5-fold increase in FBS-versus NS-treated CAL27-mTF cells). The CAL27-HPV16 cells, however, exhibited a mean difference in proliferation between NS and FBS treatments of 1.5-fold on day 1, 1.6-fold on day 2, and 2.4-fold on day 3 (Fig. 2C).

**Figure 3:**
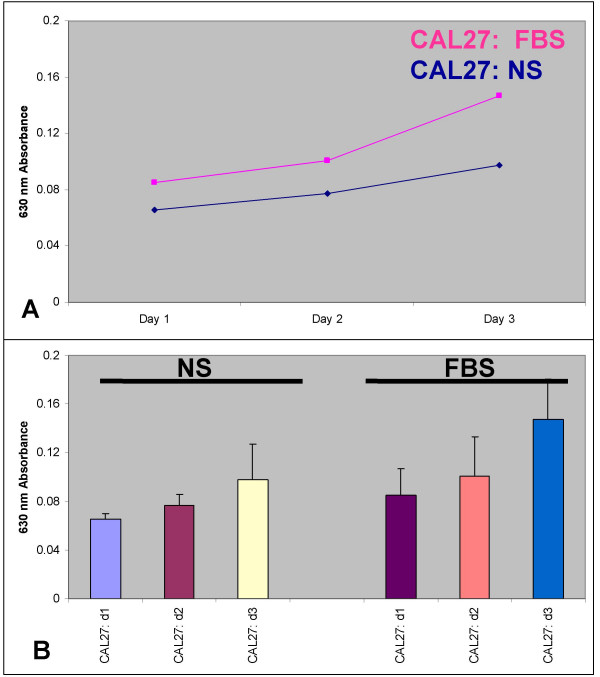
**CAL27 cells proliferated slowly when plated at low density *in vitro***. CAL27 cells plated at low density (10^4 ^cells per well) proliferated 1.5-fold over three days in the absence of serum and 1.75-fold in the presence of serum (A). FBS induced statistically significant increases in the proliferation of CAL27 cells in 96-well proliferation assays by day 3 (B) (n = 72, p < .01). Three separate, independent replications of this experiment were performed (n = 24 wells per trial).

Since the rate of proliferation for non-transfected CAL27 cells was relatively low, we performed *in vitro *proliferation assays to determine the relative, baseline levels of CAL27 cellular growth. Quiescent CAL27 cells were plated and their proliferation was measured over three days. Our results demonstrated a statistically significant increase over three days for CAL27; approximately 75% (1.75-fold) in the presence of serum (FBS) (n = 32, p < 0.01) and approximately 50% (1.5-fold) in the absence of serum (NS) (n = 32, p < 0.01). These results demonstrated that the presence of serum increased CAL27 proliferation over NS by approximately 1.5-fold, consistent with our previous observations of OSCC behavior for *in vitro *proliferation (Fig. 3) (n = 64, p < 0.01).

### Adhesion

**Figure 4:**
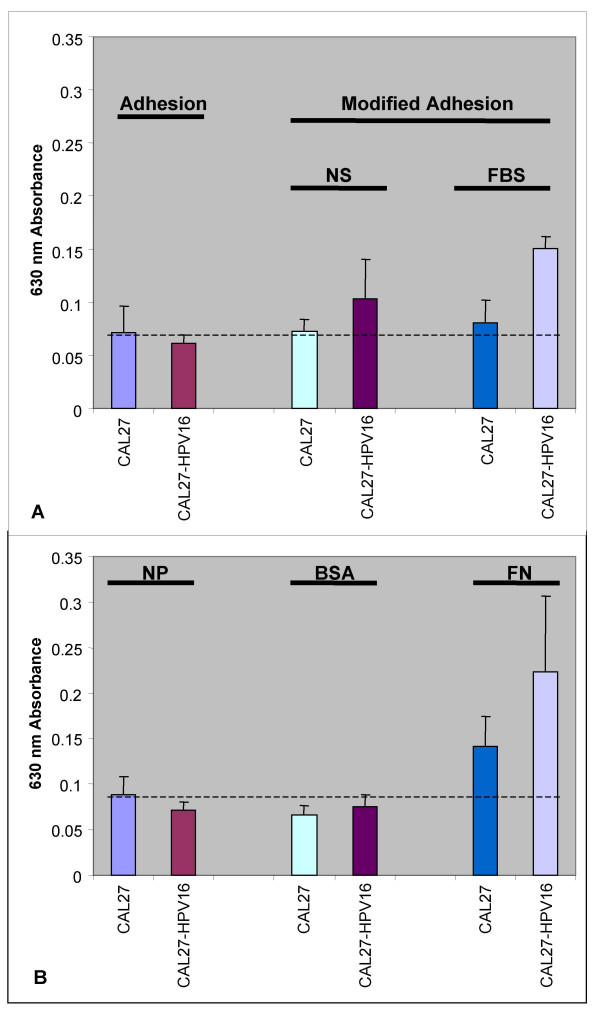
**HPV 16 transfection did not affect CAL27 adhesion *in vitro***. The presence and expression of HPV 16 did not affect CAL27 adhesion, as measured by standard 30-minute adhesion assays (A) (n = 32, p = .27). The presence and expression of HPV16 did, however, affect CAL27 adhesion in the modified adhesion assay, increasing adhesion by 43% in the absence of serum and 86% in the presence of serum in three separate, independent trials (n = 96, p < 0.01). The presence of HPV16 did not affect CAL27 adhesion in standard adhesion assays on naked plastic (NP), or wells coated with bovine serum albumin (BSA), but did increase CAL27 adhesion on fibronectin (FN) by 58% (B) (n = 24, p < 0.05).

To determine if the HPV16-stimulated increase in CAL27 proliferation was correlated with an increase in adhesion, thirty-minute *in vitro *adhesion assays were performed of CAL27 and CAL27-HPV16. Our results from the standard adhesion assay revealed that transfected CAL27-HPV16 cells did not sustain a significant increase in cellular adhesion over non-transfected CAL27 cells (n = 32, p = .27) (Fig. 4A).

To address the possibility that some adherent cells were removed during the adhesion assay procedure, particularly during plate suspension, we used a modification to this procedure which resulted in the loss of fewer adherent cells. Our results from the modified adhesion assay revealed that HPV16 increased CAL27 adhesion in the absence of serum by more than 43%. These effects on cellular adhesion were more strongly pronounced in the presence of serum, increasing adhesion of CAL27-HPV16 cells over non-transfected cells by more than 86%. The modified adhesion assay did not involve plate suspension, but only the inevitable detachment forces resulting from the fixation and staining procedure.

Our measurements indicated that approximately 20% of initial cells remained at the conclusion of standard adhesion assays. Furthermore, the modification of the adhesion assay increased only the adhesion of CAL27-HPV16 cells; 1.4-fold in the absence of serum and 1.8-fold in the presence of serum (n = 72, p < 0.01). In addition, approximately 32% of CAL27-HPV16 cells that were lost in the standard adhesion assay were retained in the NS modified adhesion assay, while 47% were retained in the FBS modified adhesion assay.

To determine the specificity of this response, we measured CAL27 adhesion to the extracellular matrix (ECM). Using the standard adhesion assay, CAL27 adhesion to naked plastic (NP), 1% bovine serum albumin (BSA), and fibronectin (FN) were measured. Our results verified that non-specific adhesion remained constant between CAL27 and CAL27-HPV16 cells (Fig. 4B). Intriguingly, the coating of wells in the adhesion assay with FN increased CAL27 adhesion 1.7-fold (nearly 35% of total cells adhered), while CAL27-HPV16 adhesion increased 2.8-fold (nearly 56% of total cells adhered)(n = 96, p < 0.05), demonstrating a statistically significant difference between CAL27 and CAL27-HPV16 cells on FN (n = 16, p < 0.05).

### Viability and morphology

Based upon our *in vitro *observations, we hypothesized that HPV16 has a direct effect on the proliferation and adhesion of CAL27 cells. Because cellular adhesion to the ECM may influence proliferation, we sought to examine further how the effects of HPV may alter other cellular phenotypes, both quantitatively and qualitatively. To this end, we examined viability of CAL27, CAL27-mTF and CAL27-HPV16 cells, estimating the number of viable cells in each assay. Our results demonstrated that between 73 and 84% of cells were viable from each treatment category used for the adhesion and proliferation assays, day 0 (Table 1). These small differences in viability did not represent statistically significant differences among these treatments (n = 212, p = .48). In addition, our results demonstrated that 97–99% of cells observed from all treatment groups were viable on day 3, and were also not statistically different (n = 561, p = .88).

**Table 1:** Trypan cell-viability assay

Cell Line	Day	Treatment	Total # cells	# viable	# non-viable	% viable
CAL27	Day 0	NS	85	62	23	73
CAL27	Day 0	FBS	85	62	23	73
CAL27-mTF	Day 0	NS	54	44	10	81
CAL27-mTF	Day 0	FBS	54	44	10	81
CAL27-HPV16	Day 0	NS	73	61	12	84
CAL27-HPV16	Day 0	FBS	73	61	12	84
CAL27	Day 2	NS	68	66	2	97
CAL27	Day 2	FBS	101	100	1	99
CAL27-mTF	Day 2	NS	76	74	2	97
CAL27-mTF	Day 2	FBS	42	42	0	100
CAL27-HPV16	Day 2	NS	105	103	2	98
CAL27-HPV16	Day 2	FBS	178	176	2	99

To determine qualitative effects of HPV transfection of CAL27 cells, we examined cultured CAL27 and CAL27-HPV16 cells to estimate the proportion of spreading to non-spreading cells. Our data demonstrated no significant differences between CAL27 and CAL27-HPV16 cells in the percent spreading in the 30-minute adhesion assay. However, CAL27-HPV16 cell morphology was significantly altered by day 1 of the proliferation assay compared with CAL27 non-transfected controls (Table 2). Our results of the NS trials demonstrated a ratio of 1:2 (spread:non-spread) for CAL27 cells, and a ratio of nearly 50:1 (spread:non-spread) for CAL27-HPV16 cells at day 1. Our results of the FBS trials demonstrated a ratio of 3:1 (spread:non-spread) for CAL27 cells and nearly 60:1 (spread:non-spread) for CAL27-HPV16 cells.

**Table 2:** Relative number and ratio of spreading cells.

Cell Line	Assay	Treatment	Total # cells	# spread	% spread	Ratio spread:non-spread
CAL27	Adhesion	N/A	45	0	0	N/A
CAL27-HPV16	Adhesion	N/A	27	0	0	N/A
CAL27	Proliferation	NS – day 1	23	7	30	1:2
CAL27-HPV16	Proliferation	NS – day 1	94	92	98	46:1
CAL27	Proliferation	FBS – day 1	63	47	75	3:1
CAL27-HPV16	Proliferation	FBS – day 1	117	115	98	58:1
CAL27	Proliferation	NS – day 3	86	31	36	1:2
CAL27-HPV16	Proliferation	NS – day 3	>300	>300	100	>300:1
CAL27	Proliferation	FBS – day 3	64	47	73	3:1
CAL27-HPV16	Proliferation	FBS – day 3	>400	>400	100	>400:1

Our data show similar trends at day 2 and day 3. At day 3, for example, the ratio of spread to non-spread CAL27 non-transfectants remained constant at 1:2, while the ratio for CAL27-HPV16 transfectants increased to over 300:1 in the absence of serum (NS). Similarly, our results demonstrated that in the presence of serum (FBS), the ratio of spread to non-spread CAL27 remained at 3:1, while the ratio for CAL27-HPV16 cells increased to more than 400:1 (Table 2).

**Figure 5:**
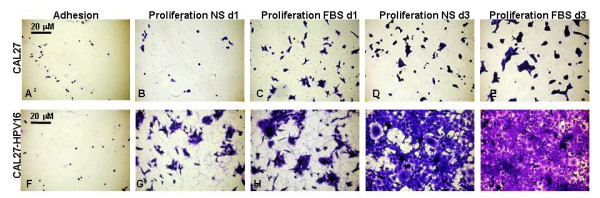
**Effects of HPV 16 on cell spreading *in vitro***. CAL27 cells from adhesion and proliferation assays (A-E) and CAL27-HPV16-transfected cells (F-J) were cultured in media containing NS-no serum (B,D,G,I) or FBS-fetal bovine serum (C,E, H, J). Cells were fixed with formalin and stained with crystal violet. Analysis revealed that HPV16+ cells increased in number and ratio of spreading under FBS treatment (H, J) and under NS treatment (G, I) to a greater extent than non-transfected cells under FBS (C, E) or NS treatments (B, D). No measurable differences in cell number or morphology were apparent in the 30-minute adhesion assays (A, F).

The phenotypic changes described in these experiments were observable between non-transfectant controls (Fig. 5A–E) and HPV16 transfectants (Fig. 5F–J). Although basic differences in cell number were observed between the treatment of cells with and without serum (Fig. 5B–C; 5D–DE), the more obvious differences in increased spreading and absolute cell number among HPV16-transfectants were evident (Fig. 5G–H; 5I–J).

### Density-dependent proliferation

**Figure 6:**
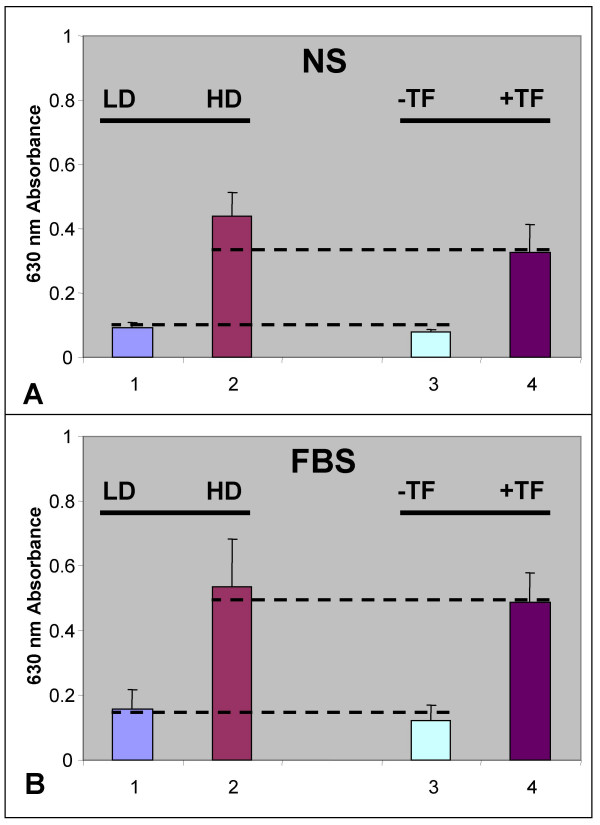
**Comparison of CAL27 density-dependent proliferation to low density CAL27-HPV16 *in vitro***. CAL27 cells plated at high density (A:2) proliferated more rapidly than those plated at low density (A:1) without serum (NS) by day 2. Cal27 cells plated at high density (B:2) also proliferated more rapidly than those plated at low density (B:1) with fetal bovine serum (FBS) by day 2. Low density proliferation of CAL27 (A:3; B:3) was increased by transfection with HPV16 (A:4-NS, B:4-FBS) to levels that approximated high density proliferation. A,B 1,3,4 = LD (low density), A,B 2 = HD (high density), A,B 4 = HPV16TF (transfected).

Our previous studies with OSCC proliferation *in vitro *revealed that CAL27 cells proliferated more rapidly when plated at higher densities (10^5 ^cells/well versus 10^4 ^cells/well), under both FBS and NS treatments, nearly doubling their proliferation even in the absence of serum (unpublished). To contextualize our results from the current study, we compared HPV16-transfected CAL27 cells to our previous studies which had demonstrated that CAL27 proliferation was density-dependent (Fig. 6). Comparative analysis of the data revealed that HPV16 transfection of CAL27 significantly increased their proliferation at low density to approximately 75% of the rates we previously observed for the proliferation of non-transfected CAL27 at high density. CAL27 cells plated at high density (Fig. 6, A2) proliferated more rapidly than those plated at low density (Fig. 6, A1) without serum (NS) by day 2. In the presence of serum, CAL27 cells plated at high density (Fig. 6, B2) also proliferated more rapidly than at low density (Fig. 6, B1) by day 2. Transfection of CAL27 with HPV16 increased low density proliferation (Fig. 6, A3; B3) to levels that approximated high density proliferation rates under both conditions (Fig. 6, A4-NS, B4-FBS). These data were consistent for both FBS and NS treatments, demonstrating an increase in proliferation of CAL27-HPV16 cells to 75% of the high density level without serum and to 95% of the high-density level in the presence of serum (Fig.6).

## Discussion

HPV E6 and E7 genes code for proteins that are capable of binding to, and inactivating, tumor suppressors, such as *Rb *and *p53 *in cervical cancers [5,11,12]. Recent reports of HPV infection in a number of oral cancers suggest that HPV may also function in a similar way in transforming normal oral mucosa [13]. The absence of HPV in premalignant lesions, combined with the lower prevalence of HPV in oral cancers, however, indicates that HPV may act as a mediator of phenotypic changes in OSCC, rather than being the etiological factor inducing initial transformation [18].

Consequently, we hypothesized that HPV16 infection mediates phenotypic alterations in OSCC cells, particularly proliferation, adhesion and morphology, and that qualitative and statistically significant quantitative differences could be identified. This study employed the well-characterized OSCC cell line, CAL27, to analyze the ability of HPV16 infection to alter these phenotypes. Notably, this study found that HPV16 infection altered the CAL27 proliferative phenotype, dramatically increasing CAL27 cell growth *in vitro*. The quantitative measurements of proliferation determined that HPV-infection increased proliferation by more than 5-fold over non-transfected cells.

This study collected some of the first evidence that HPV16 induces these proliferative changes in tandem with effects on cellular adhesion. We have demonstrated with a modified adhesion assay that HPV16 may alter adhesion, but at levels not distinguishable from the standard adhesion assay protocol. Furthermore we have provided evidence that transformed oral epithelia transfected with HPV16 have increased adhesion to a specific ECM substrate, fibronectin. These results concur with previous research which detected that HPV-immortalized genital epithelial cells and HPV-containing carcinoma cell lines expressed higher levels of intracellular and extracellular fibronectin [19,20]. These studies also found an upregulation of pp125 FAK (focal adhesion kinase), a cytoplasmic protein kinase that becomes active after cellular attachment to specific ECM proteins, such as fibronectin [21,22].

In addition to demonstrating increased proliferation, this study revealed that HPV16 also induced spreading of cells, even under low density conditions. These results, when combined with our data regarding the ECM-specific adhesion of CAL27-HPV16 on fibronectin, may represent a possible mechanism that can explain, in part, the HPV-mediated phenotypic changes, such as cell spreading, and the observed changes in proliferation and adhesion.

Our results are consistent with several lines of published evidence that suggest that HPV16 induces phenotypic changes that relate directly to progression through the cell cycle [9,11-13,23]. Specifically, the results of our research indicate that HPV16 may drive proliferation of CAL27, even when the cells are at low density. These observations may be correlated with increased ECM production, such as fibronectin, thereby providing a substrate-specific response that increases the proliferative potential of this particular OSCC under certain conditions.

These results suggest that a clear understanding of the role and contributions of infections, such as HPV, may provide new insights into the transformation and proliferative potential of OSCC. Further analysis of both the events that trigger and sustain proliferation in OSCC, and the mitigating effects of HPV infection on these existing types of cancers, may help in the elucidation of the role of HPV in determining the parameters of proliferative potential. Moreover, these results do not negate the possibility that HPV may play multiple roles in oral cancer; potentially inducing a transformation of the normal oral mucosa in some cases, and alternatively generating phenotypic changes to existing OSCC, which were already transformed due to other etiological factors, such as smoking or alcohol.

## Conclusion

Our results demonstrate that HPV16 may be a factor that influences the proliferative potential of OSCC *in vitro*. Most notably, this study represents one of the first *in vitro *analyses of the effect of HPV16 infection on the proliferative potential of transformed OSCC. The influence of HPV16 on OSCC may allow already transformed oral mucosa to proliferate more rapidly than OSCC which have not been infected. Moreover, HPV16 may influence fibronectin-specific adhesion of these cells, further promoting factors that directly influence progression through the cell cycle. Future analysis of the events that trigger and sustain the underlying cellular mechanisms of proliferation, such as the production of, and specific adhesion to ECM components, may aid in the design of more effective prognostic indicators and treatments for oral cancers with concomitant HPV infections. As more such detailed analyses are conducted, the information acquired should serve to establish a rubric for generalizing the effects that HPV infection has on OSCC to other types of pre-existing cancers.

## Methods

### Cell culture

CAL27 (human squamous cell carcinoma), GH354 (human cervical adenocarcinoma), and HTB-32 (human cervical carcinoma) cell lines were obtained from American Type Culture Collection (ATCC: Manassas, VA). CAL27 cells were maintained in Dulbecco's Modified Eagle's Medium (DMEM) with 4 mM L-glutamine, adjusted to contain 3.7 g/L sodium bicarbonate and 4.5 g/L glucose, with 1% Penicillin (10,000 units/mL)-Streptomycin (10,000 μg/mL) solution and 10% fetal bovine serum (FBS) from HyClone (Logan, UT) in 75 cm^2 ^BD Falcon tissue-culture treated flasks (Bedford, MA) at 37°C and 5% CO^2 ^in humidified chambers. GH354 cells were also maintained as described with the addition of 20% FBS. HTB-32 cells were maintained in McCoy's 5A medium with 1.5 mM L-glutamine, 2200 mg/L sodium bicarbonate from ATCC, and 10% FBS.

### Cell viability

Prior to plating cells (adhesion, proliferation assays), aliquots of trypsinized cells were stained using Trypan Blue (Sigma: St. Louis, MO) and live cells were enumerated by counting the number of Trypan-blue negative cells using a VWR Scientific Counting Chamber (Plainfield, NJ) and a Zeiss Axiovert 40 inverted microscope (Gottingen, Germany). At each time point (day 1–3), several wells were processed using the Trypan stain, and live cells were enumerated using this procedure [24,25].

### Transfection

CAL27 cells were seeded in T25 cm^2 ^BD Falcon tissue-culture treated flasks in appropriate media as described above and allowed to achieve 70% confluence. Cells were then transiently transfected by adding 1 μg/mL of the full-length human papilloma virus type 16, cloned into the pBluescript SK-vector (ATCC #45113) or the full-length human papilloma virus type 18, cloned into the pBR322 vector (ATCC #45152). The transfections were performed using the Stratagene Mammalian Transfection Kit (La Jolla, CA) according to the manufacturer's recommended protocol for CaPO_4 _transfection. As part of this procedure, CAL27 cells were co-transfected with the G418-resistant control plasmid, pWLneo. Cells were then incubated at 37°C and 5% CO^2 ^in humidified chambers for 24 hrs, at which time the media was changed and the cells were incubated as described for another 24 hrs. Cells were then split 1:10 and incubated for 24 hrs, at which time they had reached 30% confluence. Transfectants were then selected using G418 antibiotic (Stratagene: La Jolla, CA), added drop wise to the culture medium at a concentration of 100 μg/mL. After selection, CAL27-HPV16 and CAL27-HPV18 were maintained in the same cell culture conditions as described above for CAL27. Mock transfectants of CAL27 (mTF) were also established by performing the aforementioned transfection procedures, but without using virus, control plasmid, or G418.

### RT-PCR

RNA was isolated from 1.5 × 10^7 ^cells of each of the experimental and control cell lines that were cultured according to the cell culture conditions described above, using ABgene Total RNA Isolation Reagent (Epsom, Surrey, UK) and the procedure recommended by the manufacture. RT-PCR was performed with the ABgene Reverse-iT One-Step RT-PCR Kit (ReadyMix Version) and a Mastercycler gradient thermocycler (Eppendorf: Hamburg, Germany) using the following primers synthesized by SeqWright (Houston, TX): HPV 16 forward primer, ATGTTTCAGGACCCACAGGA; HPV 16 reverse primer, CCTCACGTCGCAGTAACTGT; laminin-5 γ2-subunit forward primer, TGGTGATTACAGAAGCCAGAAGG; laminin-5 γ2-subunit reverse primer, GTCAGTTGACCTGAGCATACCCAT. One μg of template RNA was used for each reaction. The reverse transcription step ran for 30 min at 47°C, followed by denaturation for 2 min at 94°C. Thirty-five amplification cycles were run, consisting of 20 sec denaturation at 94°C, 30 sec of annealing at 58°C, and 6.5 min of extension at 72°C. Final extension was run for 5 min at 72°C. Reaction products were separated by gel electrophoresis using Reliant 4% agarose gels (Cambrex: Rockland, ME). Bands were visualized by UV illumination of ethidium-bromide-stained gels and captured using a Kodak Gel Logic 100 Imaging System and 1D Image Analysis Software (Eastman Kodak: Rochester, NY). Quantitation of RT-PCR band densitometry was performed using Adobe (San Jose, CA) Photoshop imaging software, Image Analysis tools.

### Proliferation assays

*In vitro *proliferation assays of CAL27 and CAL27-HPV16 were performed in the appropriate media that was either supplemented with 10% fetal bovine serum (FBS) or that contained no serum (NS) in Corning Costar 96-well assay plates (Corning, NY). Assays were performed at two concentration; 1.2 × 10^4 ^cells per well (low density, LD) and 1.2 × 10^5 ^cells per well (high density, HD), and their proliferation was measured over three days. Cultured cells were fixed after 24 hrs (Day 1), after 48 hrs (Day 2), and after 72 hrs (Day 3) using 50 μL of 10% buffered formalin, and were subsequently stained using crystal violet 1% aqueous solution (Fisher Scientific: Fair Lawn, NJ). The relative absorbance was then measured at 630 nm using a Bio-Tek ELx808 microplate reader (Winooski, VT). Data were analyzed and graphed using Microsoft Excel (Redmond, WA). Three separate, independent replications of this experiment were performed. In addition, the number and percent of spreading and non-spreading cells were determined by visual inspection using a Zeiss Axiovert 40 inverted microscope (Gottingen, Germany) and confirmed with digital image capture and Adobe Photoshop (San Jose, CA) Image Analysis tools.

### Adhesion assays

Cell adhesion assays of CAL27 and CAL27-HPV16 were performed as previously described [26,27] in Corning Costar 96-well assay plates (Corning, NY) at a concentration of 1.2 × 10^5 ^cells per well (100 μL of 1.2 × 10^6 ^cells/mL solution) suspended in serum-free DMEM with no additives. Wells were either uncoated, (NP = naked plastic) or coated with 1% bovine serum albumin (BSA) in 10% non-fat dried milk solution or 20 μg/mL of fibronectin solution for 1 hour (60 min.) at 25°C. Cells were then plated and allowed to attach for 30 minutes at 37°C. For the standard adhesion assay, non-adherent cells were removed by suspending plates upside down in a rotating tank of 1X PBS for 10 minutes at room temperature, 25°C. Adherent cells were then fixed using 50 μL of 10% buffered formalin and subsequently stained using crystal violet 1% aqueous solution (Fisher Scientific: Fair Lawn, NJ). For the modified adhesion assay, cells were allowed to attach for 30 minutes at 37°C and were subsequently fixed using 50 μL of 10% buffered formalin without the plate suspension. The relative absorbance was then measured at 630 nm using a Bio-Tek ELx808 microplate reader. Data were analyzed and graphed using Microsoft Excel (Redmond, WA). Additionally, the number and percent of spreading and non-spreading cells were determined and confirmed in the same manner as for proliferation assays as described above.

### Statistics

The differences between treatments were measured using a *t *distribution, α = .05. All samples were analyzed using two-tailed *t *tests as departure from normality can make more of a difference in a one-tailed than in a two-tailed *t *test. As long as the sample size is even moderate (>20) for each group, quite severe departures from normality make little practical difference in the conclusions reached from these analyses [28].

## Competing interests

The author(s) declare that they have no competing interests.

## Authors' contributions

KK conceived, monitored, and coordinated the experimental design. SO and DJ carried out the microscopy, proliferation and adhesion assays. Both KK and SO contributed equally to the writing of this manuscript.
